# Supplementary material for: Lamin B loss in nuclear blebs is rupture dependent whereas increased DNA damage is rupture independent
Source: J Cell Sci. 2025 Nov 13;138(21):jcs263945. doi: 10.1242/jcs.263945 (PMC12669966; doi:10.1242/jcs.263945)
Supplement: Supplementary information [file joces-138-263945-s1.pdf]

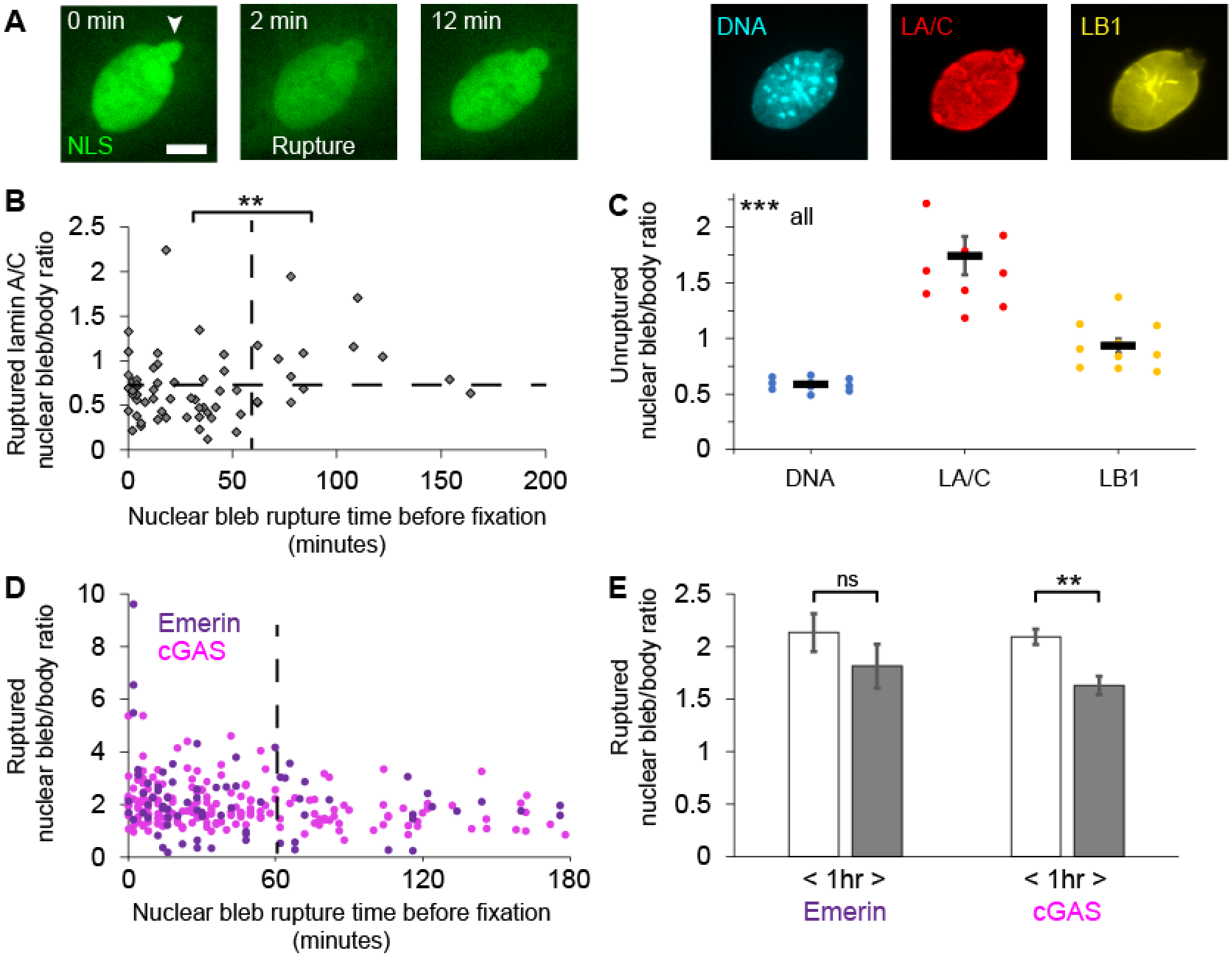

Fig. S1. Lamin A/C variable levels in nuclear blebs is due to time since last nuclear rupture. (A) Example images of MEFs from time lapse imaging via NLS-GFP and following immunofluorescence of the same nucleus stained for DNA via Hoechst (cyan), lamin A/C (red), and lamin B1 (yellow). (B) Graph of ruptured nuclear bleb/body ratio for lamin A/C relative to time elapsed since last rupture before fixation. Nuclear blebs that ruptured less than 1 hour ago were significantly depleted in 66% of cases (n = 33/50) while nuclear blebs that ruptured greater than 1 hour ago were only depleted in 36% of cases (n = 5/14). (C) Nuclear blebs that did not rupture nuclear bleb/body ratios (n = 9). (D) Scatter plot and (E) bar graph averages for less than and greater than 1 hour for ruptured nuclear bleb/body ratio relative to ruptured time for emerin and cGas (n = 72 and 81 respectively). Two-tailed (B, E) unpaired and (C) paired Student's t-test p values reported as a significance (***p < 0.001 , **p < 0.01, *p < 0.05). Scale bar = 10 μm.

Journal of Cell Science • Supplementary information

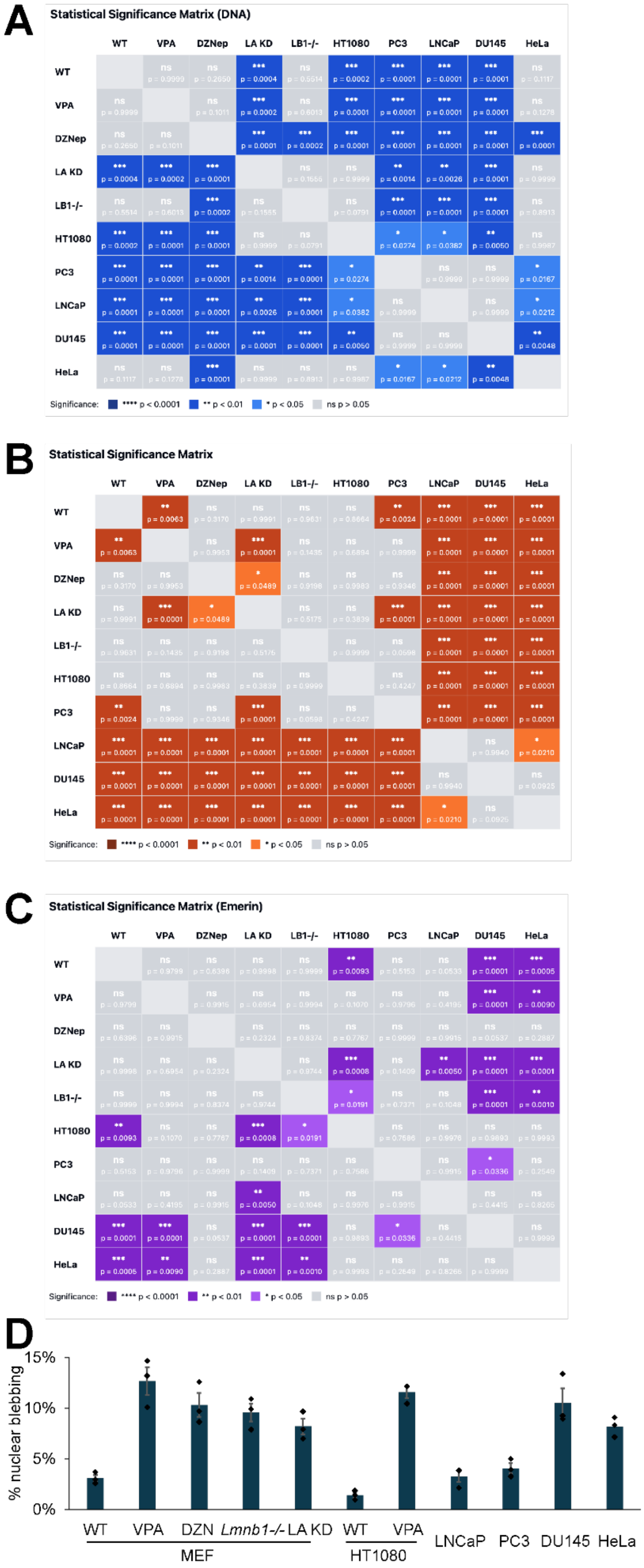

Journal of Cell Science • Supplementary information

Fig. S2. Statistical analysis of nuclear bleb composition across cell types and perturbations. Complete statistical significance matrices from one-way ANOVA with Tukey's post-hoc test for nuclear bleb/body ratios of DNA (A), lamin B (B), and emerin (C) across all experimental conditions. Analysis includes MEF wild type (WT), chromatin perturbations (VPA, DZNep), lamin perturbations (LA KD, LB1-/-), and human cancer cell lines (HT1080, PC3, LNCaP, DU145, HeLa). Each panel displays results as a heat map where color intensity corresponds to significance level: DNA (blue shades), lamin B (orange shades), and emerin (purple shades), with darker shading indicating higher significance (***$p < 0.001$, **$p < 0.01$, *$p < 0.05$), while grey indicates non-significant comparisons (ns, $p > 0.05$). Each matrix element shows the exact p-value for the corresponding pairwise comparison between conditions. (D) Graph of nuclear blebbing percentages for each cell type and/or condition. Biological triplicates where each replicate $n > 100$ cells each.

Journal of Cell Science • Supplementary information

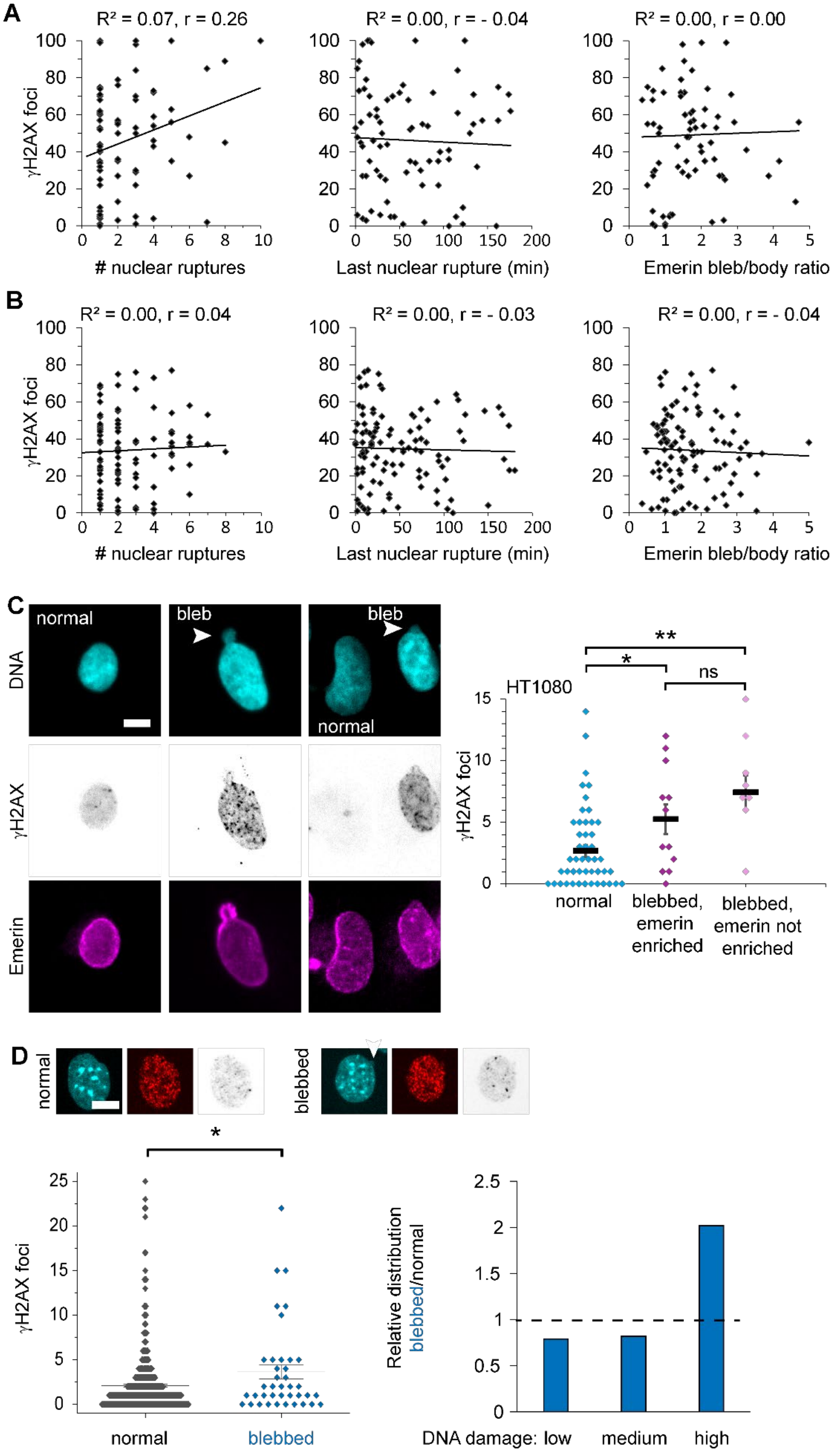

Journal of Cell Science • Supplementary information

Fig. S3. DNA damage association is independent of multiple nuclear rupture variables. Scatter plot of (A) MEF WT or (B) MEF VPA-treated nuclei γH2AX foci per number of nuclear ruptures, time since last nuclear rupture, and emerin bleb/body ratio along with R^2 and r Pearson correlation. (C) Example images of HT1080 nuclei normally shaped or blebbed stained for DNA via Hoechst (cyan), emerin (magenta), and DNA damage foci labeled by γH2AX (n = 47, 24, 7 nuclei respectively). (D) HT1080 FUCCI cells were used to measure γH2AX foci in normal and blebbed nuclei while in the G1 cell cycle via presence of CDT1 and absence of Geminin fluorescence. Graph of normal nuclei (n = 485) vs. blebbed nuclei (n = 39) number of γH2AX foci. Graphs of γH2AX DNA damage low (0-1 focus), medium (2-3 foci), and high (≥4 foci). Two-tailed unpaired Student's t-test p values reported as * < 0.05, ** < 0.01, *** < 0.001, no asterisk denotes no significance, p > 0.05. Mean ± s.e.m is graphed. Scale bar = 10 μm.
